# The Genetics of Febrile Seizures

**DOI:** 10.15844/pedneurbriefs-29-12-1

**Published:** 2015-12

**Authors:** Dipak Ram, Richard Newton

**Affiliations:** 1 Department of Paediatric Neurology, Royal Manchester Children's Hospital, Manchester, UK

**Keywords:** Febrile seizures, Epilepsy, Twin studies

## Abstract

Investigators from Virginia Commonwealth University, Norwegian Center for Epilepsy and University of Southern Denmark carried out twin studies to analyse the genetic influence of developing epilepsy after febrile seizures.

Investigators from Virginia Commonwealth University, Norwegian Center for Epilepsy and University of Southern Denmark carried out twin studies to analyse the genetic influence of developing epilepsy after febrile seizures. The subjects analysed were twin pairs that had a history of febrile seizures and data were obtained using twin registries and validated questionnaires. Febrile seizures were documented in 1051 twins (900 pairs). 61% had simple febrile seizures, 12% had complex febrile seizures, 7% had febrile status epilepticus and the remainder were unclassified. 78 twins were found to have developed epilepsy. Amongst these, the highest rates of epilepsy (22.2%) were found in those who had febrile status epilepticus, which was the smallest group. In the simple, complex and unclassified groups, the risk of developing epilepsy was 2.6%, 12% and 14.2% respectively. The authors concluded that twins with complex febrile seizures and febrile status epilepticus are at an increased risk of developing epilepsy. Amongst monozygotic twin pairs, 50 subjects who had a febrile seizure and subsequent epilepsy had a cotwin with febrile seizures. There were 28 dizygotic twins who were concordant. In both groups, the concordance rate was found to be highest in those with simple febrile seizures. The authors therefore infer that having simple febrile seizures increases the familial risk of seizures. [1]

COMMENTARY. The cause of febrile seizures is multifactorial in nature and whereas there is increasing evidence for susceptibility genes, we know no single gene to be responsible. Identification of genetic mutations has been successful in certain groups of children prone to recurrent febrile seizures, particularly those with SCN1A mutations often associated with family members with Dravet syndrome and Generalised Epilepsy with Febrile Seizures Plus (GEFS+) [2, 3]. This twin study does not state whether the grouping of the children into different febrile seizure groups (simple, complex, status etc) is defined by their first seizure or not. This is an important omission as many children will have different or at least indistinguishable seizure types during their febrile seizure career. It is seizure-type which defines recurrence and future epilepsy risk.

Some retrospective studies have shown that adults with temporal lobe epilepsy have a history of complex febrile seizures or febrile status epilepticus [4]. However, inferring a direct causal link remains controversial. A different hypothesis suggests that the seizures may have occurred due to a pre-existing hippocampal abnormality, caused by a genetic predisposition or earlier insults [5]. Recent prospective outcome studies of febrile status epilepticus have shown contradictory results, necessitating further research. This study adds to the current literature by demonstrating that there may be a strong genetic component even for simple febrile seizures. The opportunity for further research is offered by the approach of low-cost complete genome sequencing. This should add to our understanding of the interaction between genome and environment and associated epigenetic mechanisms.
